# Surgical outcomes of trabecular micro-bypass minimally invasive glaucoma surgery devices in eyes with high myopia: a 24-month retrospective multicenter cohort study

**DOI:** 10.1186/s40662-026-00512-1

**Published:** 2026-09-29

**Authors:** Bryan Chin Hou Ang, Natalie Shi Qi Wong, Aditi Kochar, Samantha Lor, Lee Ching Phang, Hla Myint Htoon, Jeremy Tan, Mitchell Lawlor, Prasanna Venkatesh Ramesh, Younhea Jung, Zhu Li Yap, Si Rui Ng, Rachel S. Chong

**Affiliations:** 1 Department of Ophthalmology, Tan Tock Seng Hospital, National Healthcare Group Eye Institute, Singapore, Singapore; 2 Yong Loo Lin School of Medicine, National University of Singapore, Singapore, Singapore; 3 Lee Kong Chian School of Medicine, Nanyang Technological University, Singapore, Singapore; 4 Department of Ophthalmology, Mayo Clinic, Jacksonville, FL USA; 5 Center for Sight, Delhi, India; 6 Singapore Eye Research Institute, Singapore, Singapore; 7 Save Sight Institute, Faculty of Medicine and Health, University of Sydney, Sydney, Australia; 8 Mahathma Eye Hospital, Trichy, India; 9 College of Medicine, Yeouido St. Mary’s Hospital, The Catholic University of Korea, Seoul, Republic of Korea; 10 Singapore National Eye Center, 11 Third Hospital Ave, Singapore, 168751 Singapore; 11 Eye Academic Clinical Program, Duke-NUS Medical School, Singapore, Singapore

**Keywords:** Myopia, Glaucoma, Open-angle, Ocular surgery

## Abstract

**Background:**

To describe outcomes of two trabecular bypass minimally invasive glaucoma surgery (MIGS) devices in highly myopic (HM) eyes with glaucoma up to 24 months postoperatively.

**Methods:**

A retrospective, observational multicenter study of HM eyes (phakic refractive spherical equivalent ≤ −6 D and/or axial length ≥ 26 mm) undergoing trabecular bypass MIGS between January 2019 and December 2023. Changes in intraocular pressure (IOP), anti-glaucoma medications and visual acuity, and rates of complications including hyphema, peripheral anterior synechiae and significant IOP elevation, defined as ≥ 30 mmHg or an increase of ≥ 6 mmHg from preoperative IOP, were recorded. IOP outcome criteria and surgical success were reported per European Glaucoma Society and American Academy of Ophthalmology (AAO) guidelines, respectively. Linear mixed models were used for continuous variables, logistic regression for categorical variables and cluster-adjusted Cox regression to compare survival between groups.

**Results:**

Two hundred and fifty-two eyes of 207 subjects across three centers were studied. One hundred and twenty-eight eyes received iStent inject and 124 received Hydrus microstent; 96.1% of iStent and 100% of Hydrus eyes underwent combined phacoemulsification. At 24 months postoperatively, ≥ 20% IOP reduction with anti-glaucoma medications (AGMs) was achieved by 35.8% (Hydrus) or 30.0% (iStent); or without AGM 13.4% (Hydrus) or 6.67% (iStent). Drop-freedom was achieved by 48.7% (Hydrus) or 38.5% (iStent). An IOP of ≤ 15 mmHg with AGM was achieved by 55.2% (Hydrus) or 63.3% (iStent); or without AGM 28.4% (Hydrus) or 21.7% (iStent). The 12-month cumulative risk of failure to maintain ≥ 20% IOP reduction with AGM was 86.7% (Hydrus) or 90.3% (iStent); or without AGM 90.1% (Hydrus) or 92.5% (iStent). Surgical success based on AAO guidelines was attained by 60.5% (Hydrus) or 53.8% (iStent) of eyes. Significant IOP elevation was the most common complication (4.84% Hydrus; 4.54% iStent), followed by hyphema (0.8% Hydrus; 4.5% iStent) at 1 month postoperatively. Outcomes did not differ significantly between the Hydrus and iStent groups.

**Conclusions:**

Both iStent inject and Hydrus microstent achieved modest IOP-lowering efficacy and low complication rates in HM eyes. Neither device exceeded the minimal clinically important difference proposed for MIGS combined with cataract surgery.

**Supplementary Information:**

The online version contains supplementary material available at https://doi.org/10.1186/s40662-026-00512-1.

## Background

Glaucoma remains the leading cause of irreversible blindness worldwide [1]. Amongst the numerous treatment modalities for glaucoma, minimally invasive glaucoma surgery (MIGS) has seen rapid adoption in recent years. Compared with traditional incisional glaucoma surgeries such as trabeculectomy and tube shunt surgery, many MIGS procedures use an ab-interno approach that minimizes collateral tissue trauma, resulting in better safety profiles while providing at least modest intraocular pressure (IOP) reduction [2]. Trabecular bypass MIGS such as the iStent series (Glaukos Corporation, CA, USA) and the Hydrus microstent (Ivantis, CA, USA) enhance the physiological aqueous outflow through the angle and are indicated for mild-to-moderate primary open-angle glaucoma (POAG) [3, 4], while also demonstrating good outcomes in ocular hypertension (OHT) [5].

Myopia, a well-known risk factor for POAG [6], is the second leading cause of severe vision impairment globally [7]. Studies have shown that eyes with moderate-to-high myopia of −3.0 dioptres (D) or greater and an IOP of ≥ 20 mmHg are almost three times more likely to have POAG than non-myopic eyes [8]. This may be attributed to structural changes in highly myopic (HM) eyes, such as greater lamina cribrosa deformity in the optic nerve head and weaker scleral support, predisposing the optic nerve to glaucomatous damage [6, 9].

Anatomical differences in HM eyes may increase complication rates in both traditional incisional glaucoma surgery and MIGS [10–13]. Cases of iStent inject malpositioning and over-implantation have been reported in HM eyes [14], possibly attributable to scleral thinning, localized ectasia, and inadequate intraoperative visualization in these eyes [15]. Canal-based MIGS devices with a fixed curvature such as the Hydrus microstent may not be compatible with the larger Schlemm’s canal (SC) diameter in HM eyes [16]. Implantation of trabecular bypass MIGS devices may also be hindered by poor visualization arising from increased corneal striae during intraoperative gonioscopy, which reflect decreased corneal hysteresis in HM eyes [17].

To our knowledge, no dedicated study has examined surgical outcomes of trabecular bypass MIGS in HM eyes. We report surgical outcomes from a retrospective, observational, multicenter cohort study that explores the efficacy and safety profiles of the iStent inject and Hydrus microstent in HM eyes.

## Methods

This retrospective, observational, multicenter cohort study involved data collection of surgeries performed between January 2019 and December 2023 from three tertiary ophthalmology centers: Mahatma Eye Hospital, Trichy, Tamil Nadu, India; Sydney Eye Surgeons, Sydney, Australia; and the Singapore National Eye Center, Singapore. The study was reviewed and approved by the Institutional Ethics Committee of Kovai Diabetes Speciality Center & Hospital, the Royal Australian and New Zealand College of Ophthalmology Human Research Ethics Committee (2020/ETH02676), and the SingHealth Centralised Institutional Review Board (2024/3272). All research adhered to the tenets of the Declaration of Helsinki.

### Study participants

Subjects were included in this study based on the following eligibility criteria: a refractive spherical equivalent of at − 6 D or more myopic prior to cataract surgery and/or an axial length of 26 mm or more; a clinical diagnosis of OHT or mild-to-moderate POAG (defined as a visual field [VF] mean deviation [MD] better than − 12 dB); and at least 12 months of postoperative follow-up. Eyes with pre-existing ocular inflammation, anterior segment dysgenesis, or peripheral anterior synechiae (PAS) were excluded from the study. All patients underwent iStent inject (iStent) or Hydrus microstent (Hydrus) implantation, with or without phacoemulsification.

### Data collection

Baseline subject demographics collected included age, sex, and ethnicity. Baseline ocular characteristics collected included diagnosis at the time of surgery, IOP measured by Goldmann applanation tonometry, best-corrected visual acuity (BCVA), refractive error, biometry, number and types of glaucoma medication, history of keratorefractive surgery, and central corneal thickness. Sixty-one of the eyes that underwent iStent implantation lacked axial length measurements but had a phakic refraction of worse than − 6 D.

Intraoperative and postoperative complications including hyphema formation, PAS formation, and significant IOP elevation, defined as ≥ 30 mmHg or an increase of ≥ 6 mmHg from preoperative IOP, were recorded over the 12 to 24-month postoperative follow-up period. Follow-up data were collected from the following postoperative timepoints: 1 month (± 14 days), 6 months (± 90 days), 12 months (± 90 days), and 24 months (± 90 days). Collected data included visual acuity, IOP, number and types of glaucoma medication, and the need for secondary IOP-lowering procedures.

### Primary outcomes

Surgical efficacy was evaluated based on the following definitions of complete success: an IOP reduction of ≥ 20% from baseline or an IOP of ≤ 15 or ≤ 18 mmHg without medication, or freedom from medication, at each timepoint. Qualified success was defined as an IOP reduction of ≥ 20% from baseline or an IOP of ≤ 15 or ≤ 18 mmHg, with medication. Safety was evaluated based on the incidence of intra- or postoperative complications including hyphema, PAS formation, significant IOP elevation, and changes in BCVA. We referenced the European Glaucoma Society guidelines for reporting IOP outcome criteria after glaucoma surgery [18]. We also examined definitions of surgical success based on international guidelines. The Glaucoma Preferred Practice Pattern Committee of the American Academy of Ophthalmology (AAO) defined the success of combined cataract surgery and MIGS as (1) either a decrease in ≥ 1 anti-glaucoma medication from baseline without an IOP increase, or an IOP of ≤ 21 mmHg with a ≥ 20% reduction from baseline without an increase in glaucoma medications, and (2) without requiring additional laser or incisional glaucoma surgery, loss of light perception vision, or hypotony. The minimal clinically important difference (MCID) for cumulative probability of success was 65% at 2 years. Treatment failure was defined as the inability to meet the above criteria across 2 consecutive postoperative visits [19].

### Statistical analysis

Patients were analyzed cross-sectionally at each follow-up timepoint. For continuous variables, the linear mixed model was used, and for categorical variables, binary logistic regression with generalized estimating equations, which accounted for inter-eye correlation. Means and standard errors were presented for continuous variables, and percentages for categorical variables. Cumulative rate of failure was assessed with Kaplan–Meier survival analysis, and a cluster-adjusted Cox regression model, which accounted for inter-eye correlation, was used to compare survival distributions between groups. Scatterplots were created to compare baseline and postoperative IOP at thresholds of 15, 18, and 21 mmHg, ≥ 20% IOP reduction, and BCVA. Missing data were handled using available case analysis. Eyes were included in the analysis at each visit if outcome data were available, regardless of whether data were missing at other timepoints. No imputation of missing values was performed. Multivariable models adjusting for prespecified covariates did not converge, owing to complete separation within the ethnicity and standalone surgery covariates (Additional file 1 Supplementary Tables 1.1–1.8). Adjusted estimates were therefore omitted, and only unadjusted odds ratios (ORs) are presented. Statistical analysis was performed using Jamovi 2.3.28.0 (the jamovi project, Sydney, Australia) [20], Stata Release 18 (StataCorp, Texas, USA) [21], and RStudio Version 2025.05.0+496 (Posit Team, Massachusetts, USA) [22]. A *P* value of less than 0.05 was considered statistically significant.

## Results

### Baseline demographics

Two hundred and fifty-two eyes of 207 subjects across three tertiary ophthalmology centers were enrolled in the study. Case distribution by center is detailed in Additional file 1 Supplementary Table 2. One hundred and twenty-eight eyes of 115 subjects received the iStent inject, while 124 eyes of 92 subjects received the Hydrus microstent. Tables 1 and 2 summarize baseline demographics. The mean baseline IOPs were 15.55 ± 0.46 mmHg and 15.19 ± 0.46 mmHg in the iStent and Hydrus groups, respectively (*P* = 0.587). The mean numbers of anti-glaucoma eyedrops in the iStent and Hydrus groups were 1.95 ± 0.12 and 1.98 ± 0.11, respectively (*P* = 0.933). The mean duration of follow-up was 17.72 ± 0.67 months in the iStent group and 18.22 ± 0.76 months in the Hydrus group (*P* = 0.619).

**Table 1:** Baseline demographics by patient (iStent versus Hydrus)

Patient demographics	iStent (n = 115)	Hydrus (n = 92)	*P* value
Age (years), mean ± SE^†^	66.04 ± 0.74 (n = 113)	67.42 ± 0.83	0.213
Mean duration of follow-up (months), mean ± SD	17.72 ± 0.67	18.22 ± 0.76	0.619
Female, no. (%)^†^	43/113 (38.1%)	25/92 (27.2%)	0.100
Race, no. (%)^‡^			
Chinese	55/105 (52.4%)	84/92 (91.3%)	**< 0.001**
Caucasian	31/105 (29.5%)	1/92 (1.1%)	
Indian	14/105 (13.3%)	5/92 (5.4%)	
Malay	1/105 (1.0%)	2/92 (2.2%)	
Others	4/105 (3.8%)	0/92 (0.0%)	

*SD* = standard deviation; *SE* = standard error

*P* values in bold indicate statistical significance

^†^n = 113 for iStent group due to missing data (2 eyes)

^‡^n = 105 for iStent group due to missing data (10 eyes)

**Table 2:** Baseline demographics by eye (iStent versus Hydrus)

Ocular characteristics	iStent (n = 128)	Hydrus (n = 124)	*P* value
Laterality, no. (%)			
Right	68/128 (53.1%)	63/124 (50.8%)	0.576
Left	60/128 (46.9%)	61/124 (49.2%)	
Diagnosis, no. (%)			
POAG	119/128 (93.0%)	116/124 (93.5%)	0.878
OHT	9/128 (7.0%)	8/124 (6.5%)	
Presence of myopic macular degeneration, no. (%)			
Yes	6/128 (4.7%)	9/124 (7.2%)	0.478
No	122/128 (95.3%)	115/124 (92.7%)	
Previous keratorefractive surgery, no. (%)			
No	126/128 (98.4%)	119/124 (96%)	0.270
LASIK	2/128 (1.6%)	5/124 (4.0%)	
Procedure, no. (%)			
Combined phacoemulsification/MIGS	123/128 (96.1%)	124/124 (100%)	NA
Standalone MIGS	5/128 (3.9%)	0/124 (0.0%)	
No. of IOP-lowering medications at baseline, no. (%)			
0	11/128 (8.6%)	2/124 (1.61%)	
1	48/128 (37.5%)	42/124 (33.9%)	
2	32/128 (25.0%)	51/124 (41.1%)	
3	18/128 (14.1%)	15/124 (12.1%)	
4	13/128 (10.2%)	14/124 (11.3%)	
5	4/128 (3.1%)	0/124 (0.0%)	
6	2/128 (1.6%)	0/124 (0.0%)	
Mean ± SE	1.95 ± 0.12	1.98 ± 0.11	0.933
Axial length (mm), mean ± SE^†^	27.20 ± 0.15 (n = 67)	27.29 ± 0.11 (n = 124)	0.643
Spherical equivalent at baseline, mean ± SE^‡^	– 6.72 ± 0.37 (n = 124)	– 8.16 ± 0.45 (n = 90)	**0.015**
Baseline BCVA (Snellen), no. (%)^§^			
6/12 and better	75/127 (59.1%)	70/124 (56.5%)	
Worse than 6/12	52/127 (40.9%)	54/124 (43.5%)	
Baseline BCVA (logMAR), mean ± SE^¶^	0.366 ± 0.029 (n = 127)	0.370 ± 0.030 (n = 116)	0.922
Visual field, mean ± SE			
Mean deviation (dB)^††^	− 6.12 ± 0.51 (n = 109)	− 8.48 ± 0.50	**0.001**
Corneal thickness (µm), mean ± SE^‡‡^	543.10 ± 3.82 (n = 127)	551.61 ± 4.38 (n = 112)	0.145
Baseline IOP on GAT (mmHg)			
Mean ± SE	15.49 ± 0.46	15.19 ± 0.46	0.587

*BCVA* = best-corrected visual acuity; *GAT* = Goldmann applanation tonometry; *IOP* = intraocular pressure; *LASIK* = laser-assisted in situ keratomileusis; *MIGS* = minimally invasive glaucoma surgery; *OHT* = ocular hypertension; *POAG* = primary open-angle glaucoma; *SE* = standard error

*P* values in bold indicate statistical significance

^†^n = 67 for iStent group due to missing data (61 eyes)

^‡^n = 124 for iStent group and n = 90 for Hydrus group due to missing data (4 and 34 eyes respectively)

^§^n = 127 for iStent group due to missing data (1 eye)

^¶^n = 127 for iStent group and n = 116 for Hydrus group due to missing data (1 eye and 8 eyes respectively)

^††^n = 109 for iStent group due to missing data (19 eyes)

^‡‡^n = 127 for iStent group and n = 112 for Hydrus group due to missing data (1 eye and 12 eyes respectively)

Most baseline variables did not differ significantly between groups, apart from ethnicity and VF MD. Chinese participants formed the ethnic majority in both the iStent and Hydrus groups, with a greater predominance in the Hydrus group (iStent: 52.4%, Hydrus: 91.3%, *P* < 0.001), while MD values were worse by approximately 2 dB in the Hydrus group (Hydrus: − 8.48 ± 0.50, iStent: − 6.12 ± 0.51 dB, *P* = 0.001). In addition, all patients in the Hydrus group underwent Hydrus implantation with phacoemulsification, while a small minority of patients in the iStent group underwent standalone implantation (combination surgery: iStent: 96.1%, Hydrus: 100%). Axial length measurements were only available for 67 out of 128 eyes in the iStent group and all eyes in the Hydrus group; the groups did not differ significantly (iStent: 27.20 ± 0.15 mm, Hydrus: 27.29 ± 0.11 mm; *P* = 0.643).

### Primary outcomes

**Fig. 1:**
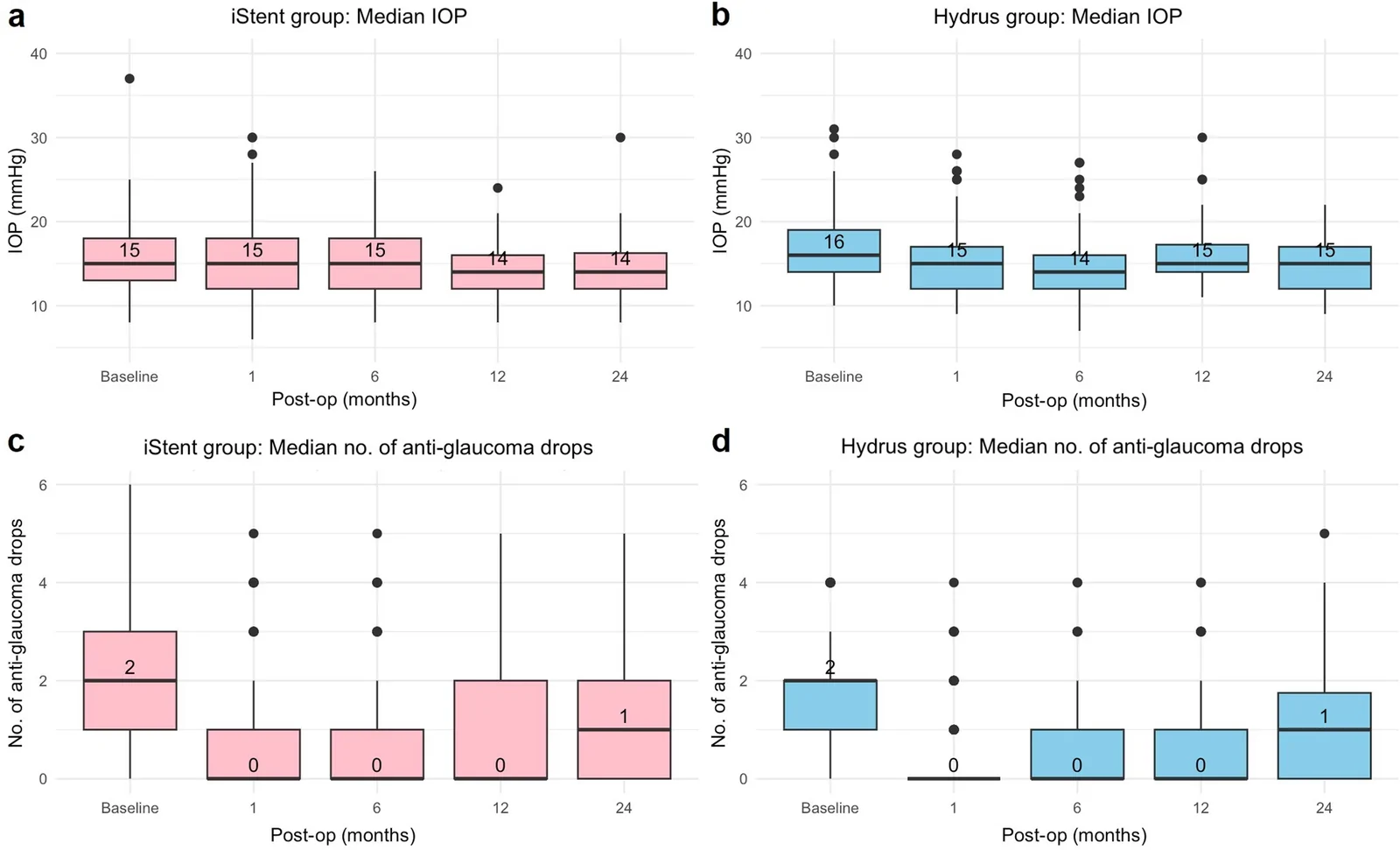
Baseline versus postoperative month 1–24 median IOPs and anti-glaucoma drops. **a** IOP for iStent; **b** IOP for Hydrus; **c** anti-glaucoma drops for iStent; **d** anti-glaucoma drops for Hydrus. IOP, intraocular pressure; Post-op, post-operation. Bars represent interquartile ranges, whiskers represent ranges, black points represent outliers

The baseline versus postoperative median IOPs and number of anti-glaucoma eyedrops, up to postoperative month (POM) 24, are depicted in Fig. 1.

#### Complete success

Complete success rates between the iStent and Hydrus groups are summarized in Table 3.

**Table 3:** Complete success (iStent versus Hydrus) in postoperative months 1–24

Outcome	POM1				POM6			
	iStent (n = 110)	Hydrus (n = 122)	Odds ratio (95% CI)	*P * value	iStent (n = 102)	Hydrus (n = 105)	Odds ratio (95% CI)	*P* value
Reduction in IOP from baseline of ≥ 20% without eyedrops (n, %)	18 (16.4%)	37 (30.3%)	0.45 (0.22–0.88)	**0.019**	13 (12.7%)	27 (25.7%)	0.42 (0.20–0.89)	**0.024**
Reduction in IOP from baseline of < 20% without eyedrops (n, %)	92 (83.6%)	85 (69.7%)	1		89 (87.3%)	78 (74.3%)	1	
IOP without eyedrops (n, %)								
≤ 15 mmHg	47 (42.7%)	62 (50.8%)	0.702 (0.37–1.32)	0.267	35 (34.3%)	51 (48.6%)	0.519 (0.26–1.02)	0.059
≤ 18 mmHg	64 (58.2%)	80 (65.6%)	0.872 (0.37–2.05)	0.753	50 (49.0%)	68 (64.8%)	0.667 (0.28–1.58)	0.356
≤ 21 mmHg	71 (64.5%)	86 (70.5%)	1.070 (0.33–3.46)	0.901	57 (55.9%)	71 (67.6%)	1.770 (0.42–7.47)	0.441
No Change	39 (35.5%)	36 (29.5%)	1					
Proportion of eyes off drops (n, %)^†^	74 (67.3%)	94/124 (75.8%)	0.657 (0.36–1.21)	0.176	63/106 (59.4%)	73/108 (67.6%)	0.703 (0.38–1.29)	0.252
No. of anti-glaucoma eyedrops								
Mean ± SD	0.80 ± 1.34	0.44 ± 0.90	NA	0.020	0.83 ± 1.27	0.56 ± 0.96	NA	0.076

Outcome	POM12				POM24			
	iStent (n = 113)	Hydrus (n = 84)	Odds ratio (95% CI)	*P * value	iStent (n = 60)	Hydrus (n = 67)	Odds ratio (95% CI)	*P * value
Reduction in IOP from baseline of ≥ 20% without drops (n, %)	19 (16.8%)	13 (15.5%)	1.03 (0.48–2.51)	0.816	4 (6.67%)	9 (13.4%)	0.47 (0.14–1.62)	0.231
Reduction in IOP from baseline of < 20% without drops (n, %)	94 (83.2%)	71 (84.5%)	1		56 (93.3%)	58 (86.6%)	1	
IOP without drops (n, %)								
≤ 15 mmHg	38 (33.6%)	31 (36.9%)	0.937 (0.48–1.85)	0.851	13 (21.7%)	19 (28.4%)	0.703 (0.31–1.67)	0.426
≤ 18 mmHg	60 (53.1%)	41 (48.8%)	1.690 (0.68–4.23)	0.267	19 (31.7%)	29 (43.3%)	0.609 (0.18–2.02)	0.417
≤ 21 mmHg	62 (54.9%)	45 (53.6%)	0.379 (0.08–2.19)	0.279	23 (38.3%)	29 (43.3%)	NA	NA
No Change	51 (45.1%)	39 (46.4%)	1		37 (61.7%)	38 (56.7%)	1	
Proportion of eyes off drops (n, %)^†^	63/114 (55.3%)	48/91 (52.7%)	1.120 (0.60–2.04)	0.745	25/65 (38.5%)	38/78 (48.7%)	0.655 (0.32–1.33)	0.241
No. of anti-glaucoma eyedrops								
Mean ± SD	0.94 ± 1.28	0.79 ± 1.05	NA	0.366	1.31 ± 1.27	0.94 ± 1.18	NA	0.086

*CI* = confidence interval; *IOP* = intraocular pressure; *POM* = postoperative month; *SD* = standard deviation

*P* values in bold indicate statistical significance

^†^Additional data were available for this variable

##### *IOP reduction of * ≥ *20% from baseline without anti-glaucoma eyedrops*

At POM1, an IOP reduction of ≥ 20% from baseline without anti-glaucoma eyedrops was achieved in 30.3% and 16.4% of eyes in the Hydrus and iStent groups, respectively (OR = 0.45, 95% CI: 0.22–0.88; *P* = 0.019); at POM6, this was achieved in 25.7% and 12.7% of eyes in the Hydrus and iStent groups, respectively (OR = 0.42, 95% CI: 0.20–0.89; *P* = 0.024); at POM12, this was achieved in 15.5% and 16.8% of eyes in the Hydrus and iStent groups, respectively (OR = 1.03, 95% CI: 0.48–2.51; *P* = 0.816); at POM24, this was achieved in 13.4% and 6.67% of eyes in the Hydrus and iStent groups, respectively (OR = 0.47, 95% CI: 0.14–1.62; *P* = 0.231). Compared with the iStent implant, the Hydrus implant showed a significantly greater proportion of eyes achieving an IOP reduction of ≥ 20% without anti-glaucoma eyedrops from POM1 to 6, but this difference was no longer statistically significant from POM12 to 24.

**Fig. 2:**
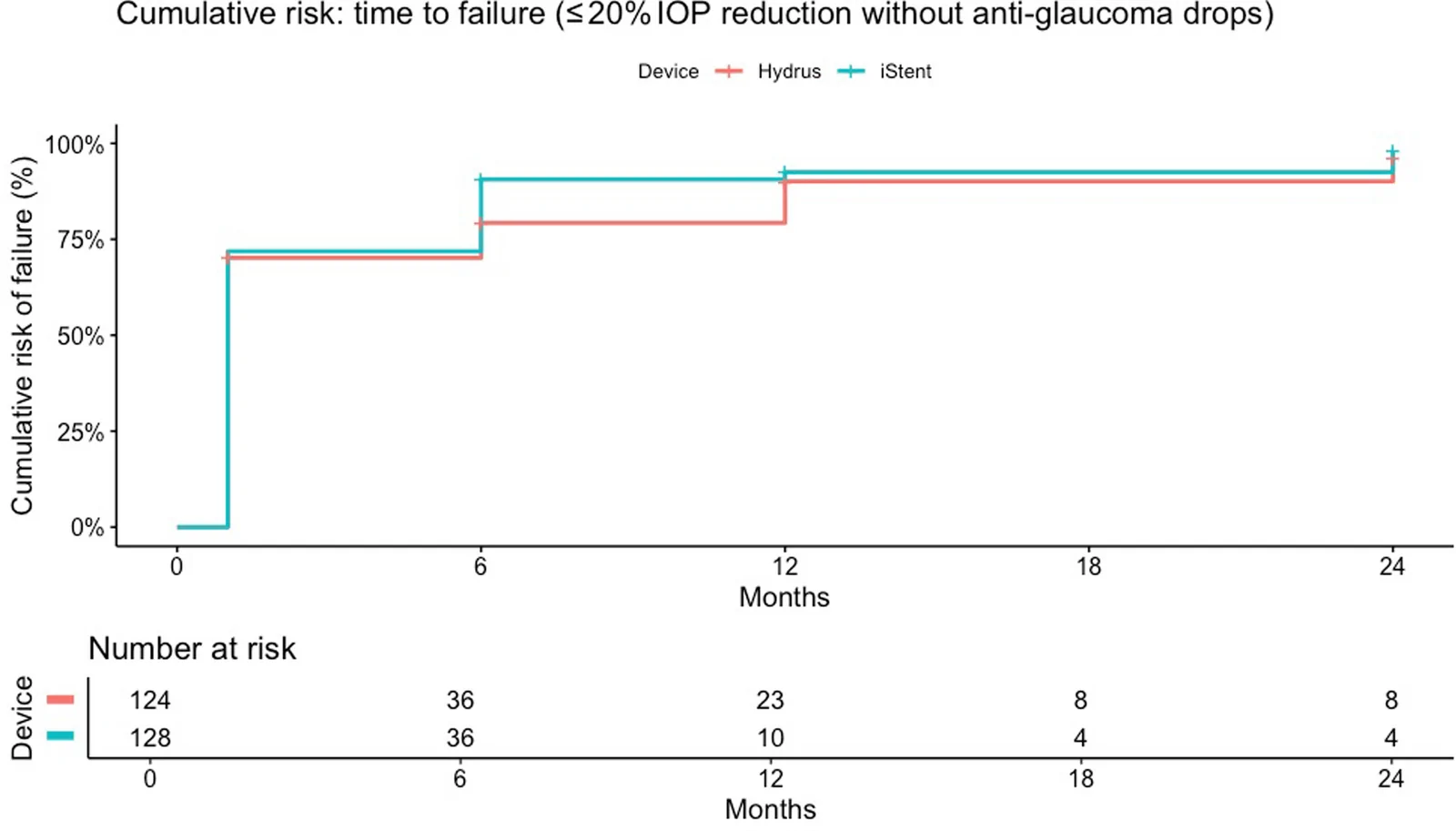
Cumulative risk of failure of maintaining ≥ 20% IOP reduction without anti-glaucoma drops, Hydrus versus iStent. IOP, intraocular pressure

Both groups had comparable rates of eyes achieving ≥ 20% IOP reduction without anti-glaucoma eyedrops after POM6. Kaplan–Meier survival analysis (Fig. 2) showed no significant differences between the two groups for time to failure of maintaining ≥ 20% IOP reduction without anti-glaucoma eyedrops (1-year cumulative risk: iStent: 92.5%, Hydrus: 90.1%; *P* = 0.224).

##### *Proportion of patients achieving drop-freedom*

Drop-freedom did not differ significantly between groups at any measured timepoint (all *P* > 0.05). At POM1, drop-freedom was achieved in 75.8% and 67.3% of eyes in the Hydrus and iStent groups, respectively (OR = 0.657, 95% CI: 0.36–1.21). At POM6, drop-freedom was achieved in 67.6% and 59.4% of eyes in the Hydrus and iStent groups, respectively (OR = 0.703, 95% CI: 0.38–1.29). At POM12, drop-freedom was achieved in 52.7% and 55.3% of eyes in the Hydrus and iStent groups, respectively (OR = 1.12, 95% CI: 0.60–2.04). At POM24, drop-freedom was achieved in 48.7% and 38.5% of eyes in the Hydrus and iStent groups, respectively (OR = 0.655, 95% CI: 0.32–1.33).

##### *Proportion of patients achieving IOP of * ≤ *15 or * ≤ *18 mmHg without anti-glaucoma drops*

The proportion of patients achieving an IOP of ≤ 15 or ≤ 18 mmHg without anti-glaucoma drops did not differ significantly between the Hydrus and iStent groups at any measured timepoint (all *P* > 0.05). At POM1, an IOP of ≤ 15 mmHg or ≤ 18 mmHg without anti-glaucoma drops was achieved in 50.8% or 65.6% of eyes in the Hydrus group, respectively, compared with 42.7% or 58.2% of eyes in the iStent group (OR = 0.702, 95% CI: 0.37–1.32; and 0.872, 95% CI: 0.37–2.05); at POM6, this was achieved in 48.6% or 64.8% of eyes in the Hydrus group, compared with 34.3% or 49.0% of eyes in the iStent group (OR = 0.519, 95% CI: 0.26–1.02; and 0.667, 95% CI: 0.28–1.58); at POM12, this was achieved in 36.9% or 48.8% of eyes in the Hydrus group, compared with 33.6% or 53.1% of eyes in the iStent group (OR = 0.937, 95% CI: 0.48–1.85; and 1.69, 95% CI: 0.68–4.23); at POM24, this was achieved in 28.4% or 43.3% of eyes in the Hydrus group, compared with 21.7% or 31.7% of eyes in the iStent group, respectively (OR = 0.703, 95% CI: 0.31–1.67; and 0.609, 95% CI: 0.18–2.02).

#### Qualified success

Qualified success rates between the iStent and Hydrus groups are summarized in Table 4.

**Table 4:** Qualified success (iStent versus Hydrus) in postoperative months 1–24

Outcome	POM1				POM6				
	iStent (n = 110)	Hydrus (n = 122)	Odds ratio (95% CI)	*P* value	iStent (n = 102)		Hydrus (n = 105)	Odds ratio (95% CI)	*P* value
Reduction in IOP from baseline of ≥ 20% with/without eyedrops (n, %)	22 (20.0%)	44 (36.1%)	0.44 (0.24–0.80)	**0.007**	26 (25.5%)		37 (35.2%)	0.63 (95% CI: 0.35–1.15)	0.129
Reduction in IOP from baseline of < 20% with/without eyedrops (n, %)	88 (80.0%)	78 (63.9%)	1		76 (74.5%)		68 (64.8%)	1	
IOP with/without eyedrops (n, %)									
≤ 15 mmHg	62 (56.4%)	71 (58.2%)	0.742 (0.30–1.85)	0.523	60 (58.8%)		68 (64.8%)	0.552 (0.14–2.21)	0.401
≤ 18 mmHg	85 (77.3%)	100 (82.0%)	0.675 (0.29–1.88)	0.452	84 (82.4%)		92 (87.6%)	0.626 (0.15–2.70)	0.529
≤ 21 mmHg	97 (88.2%)	111 (91.0%)	0.922 (0.27–3.14)	0.896	94 (92.2%)		100 (95.2%)	0.781 (0.16–3.76)	0.758
No change	13 (11.8%)	11 (9.0%)	1		8 (7.8%)		5 (4.8%)	1	
No. of anti-glaucoma eyedrops									
Mean ± SD	0.348 ± 0.045	0.244 ± 0.045	NA	0.101	0.409 ± 0.049		0.320 ± 0.051	NA	0.207

Outcome	POM12					POM24			
	iStent (n = 113)	Hydrus (n = 84)	Odds ratio (95% CI)	*P* value	iStent (n = 60)		Hydrus (n = 67)	Odds ratio (95% CI)	*P* value
Reduction in IOP from baseline of ≥ 20% with/without drops (n, %)	36 (31.9%)	17 (20.2%)	1.84 (0.95–3.57)	0.070	18 (30.0%)		24 (35.8%)	0.77 (0.36–1.61)	0.481
Reduction in IOP from baseline of < 20% with/without drops (n, %)	77 (68.1%)	67 (79.8%)	1		42 (70.0%)		43 (64.2%)	1	
IOP with/without drops (n, %)									
≤ 15 mmHg	75 (66.4%)	45 (53.6%)	6.72 (0.63–71.32)	0.114	38 (63.3%)		37 (55.2%)	2.00 (0.19–21.39)	0.565
≤ 18 mmHg	108 (95.6%)	71 (84.5%)	5.13 (0.46–56.73)	0.182	52 (86.7%)		60 (89.6%)	1.18 (0.12–11.82)	0.890
≤ 21 mmHg	112 (99.1%)	80 (95.2%)	1.76 (0.14–22.00)	0.663	59 (98.3%)		65 (97.0%)	2.69 (0.20–35.83)	0.454
No change	1 (0.9%)	4 (4.8%)	1		1 (1.7%)		2 (3.0%)	1	
No. of anti-glaucoma eyedrops									
Mean ± SD	0.445 ± 0.049	0.457 ± 0.059	NA	0.877	0.602 ± 0.063		0.514 ± 0.061	NA	0.323

*CI* = confidence interval; *IOP* = intraocular pressure; *POM* = postoperative month; *SD* = standard deviation

*P* values in bold indicate statistical significance

##### *IOP reduction of * ≥ *20% from baseline with anti-glaucoma eyedrops*

**Fig. 3:**
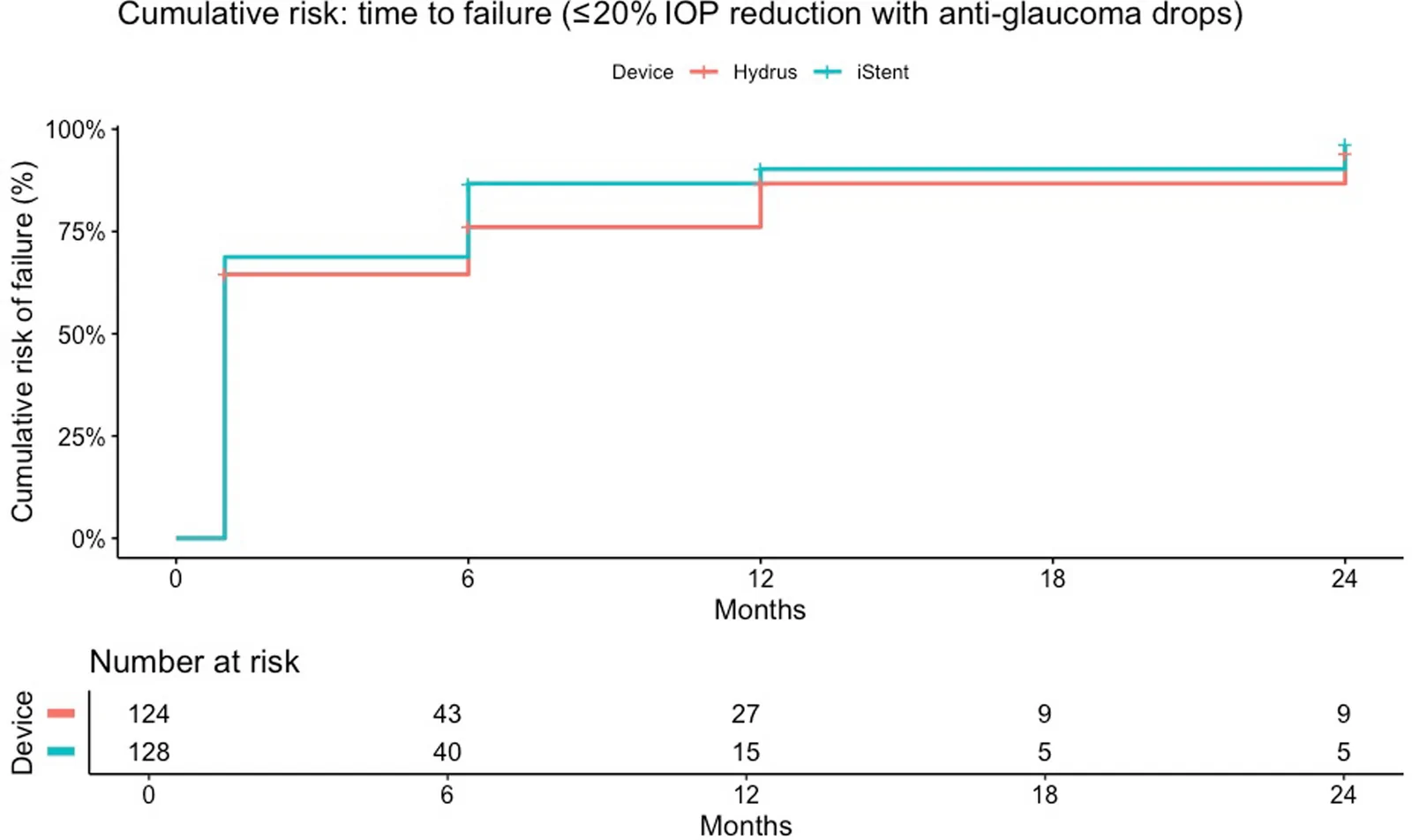
Cumulative risk of failure of maintaining ≥ 20% IOP reduction with anti-glaucoma drops, Hydrus versus iStent. IOP, intraocular pressure

At POM1, an IOP reduction of ≥ 20% from baseline with anti-glaucoma eyedrops was achieved in 36.1% and 20.0% of eyes in the Hydrus and iStent groups, respectively (OR = 0.44, 95% CI: 0.24–0.80; *P* = 0.007); at POM6, this was achieved in 35.2% and 25.5% of eyes in the Hydrus and iStent groups, respectively (OR = 0.63, 95% CI: 0.35–1.15; *P* = 0.129); at POM12, this was achieved in 20.2% and 31.9% of eyes in the Hydrus and iStent groups, respectively (OR = 1.84, 95% CI: 0.95–3.57; *P* = 0.070); at POM24, this was achieved in 35.8% and 30.0% of eyes in the Hydrus and iStent groups, respectively (OR = 0.77, 95% CI: 0.36–1.61; *P* = 0.481). Compared with the iStent implant, the Hydrus implant showed a significantly greater proportion of eyes achieving an IOP reduction of ≥ 20% from baseline with anti-glaucoma eyedrops at POM1, but no significant difference from POM6 onwards. Kaplan–Meier survival analysis (Fig. 3) showed no significant difference between the two groups for time to failure of maintaining ≥ 20% IOP reduction with anti-glaucoma eyedrops (1-year cumulative risk: iStent: 90.3%, Hydrus: 86.7%; *P* = 0.198).

##### *Proportion of patients achieving IOP of* ≤ *15 or* ≤ *18 mmHg with anti-glaucoma drops*

The proportion of patients achieving an IOP of ≤ 15 or ≤ 18 mmHg with anti-glaucoma drops did not differ significantly between the Hydrus and iStent groups at any measured timepoint (all *P* > 0.05). At POM1, an IOP of ≤ 15 mmHg or ≤ 18 mmHg with anti-glaucoma drops was achieved in 58.2% or 82.0% of eyes in the Hydrus group, respectively, compared with 56.4% or 77.3% of eyes in the iStent group (OR = 0.742, 95% CI: 0.30–1.85; and 0.675, 95% CI: 0.29–1.88); at POM6, this was achieved in 64.8% or 87.6% of eyes in the Hydrus group, compared with 58.8% or 82.4% of eyes in the iStent group (OR = 0.552, 95% CI: 0.14–2.21; and 0.626, 95% CI: 0.15–2.70); at POM12, this was achieved in 53.6% or 84.5% of eyes in the Hydrus group, compared with 66.4% or 95.6% of eyes in the iStent group (OR = 6.72, 95% CI: 0.63–71.32; and 5.13, 95% CI: 0.46–56.73); at POM24, this was achieved in 55.2% or 89.6% of eyes in the Hydrus group, compared with 63.3% or 86.7% of eyes in the iStent group, respectively (OR = 2.00, 95% CI: 0.19–21.39; and 1.18, 95% CI: 0.12–11.82).

##### *Number of anti-glaucoma eyedrops*

At POM1, the mean number of anti-glaucoma drops in the Hydrus and iStent groups was 0.348 ± 0.045 and 0.244 ± 0.045, respectively; 0.409 ± 0.049 and 0.320 ± 0.051 at POM6; 0.445 ± 0.049 and 0.457 ± 0.059 at POM12; and 0.602 ± 0.063 and 0.514 ± 0.061 at POM24. No significant difference was observed between the two groups at any timepoint (all *P* > 0.05). Both groups showed a statistically significant reduction in anti-glaucoma eyedrops from baseline at all timepoints (all *P* < 0.001), further detailed in Table 5. Scatterplots of combined success criteria (≤ 15 and ≤ 18 mmHg, ≥ 20% IOP reduction) at each timepoint for the iStent and Hydrus groups, with and without anti-glaucoma eyedrops, are depicted in Additional file 2 Supplementary Fig. 1.

**Table 5:** Reduction in number of glaucoma eyedrops post-surgery compared with baseline (iStent and Hydrus)

Comparison	Mean difference (mean ± SE)	95% CI	*P* value
iStent			
No. of eyedrops, Baseline vs. POM1	1.63 ± 0.107	1.42–1.83	**< 0.001**
No. of eyedrops, Baseline vs. POM6	1.55 ± 0.113	1.33–1.77	**< 0.001**
No. of eyedrops, Baseline vs. POM12	1.51 ± 0.111	1.29–1.72	**< 0.001**
No. of eyedrops, Baseline vs. POM24	1.34 ± 0.116	1.11–1.57	**< 0.001**
Hydrus			
No. of eyedrops, Baseline vs. POM1	1.73 ± 0.096	1.55–1.92	**< 0.001**
No. of eyedrops, Baseline vs. POM6	1.65 ± 0.103	1.45–1.85	**< 0.001**
No. of eyedrops, Baseline vs. POM12	1.50 ± 0.106	1.30–1.71	**< 0.001**
No. of eyedrops, Baseline vs. POM24	1.46 ± 0.113	1.24–1.69	**< 0.001**

*CI* = confidence interval; *POM* = postoperative month; *SE* = standard error

*P* values in bold indicate statistical significance

#### Need for further IOP-lowering procedures

Three eyes in the iStent group required further IOP-lowering procedures, including one eye requiring filtration surgery (0.91%) at POM1, one eye requiring deep sclerectomy (0.88%) at POM12, and another requiring selective laser trabeculoplasty (1.54%) at POM24. All three eyes underwent combined phacoemulsification with iStent implantation. None of the eyes in the Hydrus group required further IOP-lowering procedures at any timepoint.

#### Surgical outcomes based on international guidelines

Forty-nine out of 81 eyes (60.5%) in the Hydrus group and 35 out of 65 eyes (53.8%) in the iStent group met the criteria for surgical success, based on the AAO guidelines, at 2 years [19]. The difference between the two groups was not statistically significant (*P* = 0.440).

### Secondary outcomes

Postoperative complication rates for the iStent and Hydrus groups are detailed in Additional File 1 Supplementary Tables 3 and 4. Overall, the Hydrus group appeared to have lower complication rates than the iStent group. The Hydrus group had lower rates of hyphema formation than the iStent group (POM1 0.8% vs. 4.5%; POM6 0.0% vs. 2.8%, respectively), but these rates were too low to determine statistical significance. Rates of significant IOP elevations (POM1 4.84% vs. 4.54%; POM6 1.82% vs. 1.89%; POM12 1.1% vs. 0%; POM24 2.5% vs. 1.5%, respectively) and rates of PAS formation (POM1 0.81% vs. 0.91%; POM6 0.91% vs. 0.94%; POM12 1.10% vs. 0.88%, respectively) were similar between the Hydrus and iStent groups. No other complications apart from significant IOP elevations were noted at POM24. Additional file 2 Supplementary Fig. 2 shows a scatterplot of BCVA at baseline and POM12. In the iStent group, one patient (0.885%) had a significant BCVA worsening of ≥ 0.2 logMAR, while none of the eyes in the Hydrus group experienced any significant BCVA reduction. Additional file 1 Supplementary Table 5 compares BCVA between the iStent and Hydrus groups and shows significantly better BCVA in the iStent group at POM6–24.

## Discussion

To the best of our knowledge, this is the first study to demonstrate that in HM eyes, the iStent inject and Hydrus microstent, both trabecular bypass MIGS devices, have similar efficacy with low complication rates up to POM24. The Hydrus microstent tended to perform better in early POMs, while the iStent implant tended to perform better in the later POMs for both complete and qualified success criteria. By POM24, the two groups no longer differed for any success criterion. The number of anti-glaucoma drops did not differ statistically between the two groups at any timepoint.

Rates of complications, including hyphema, significant IOP elevation, and PAS formation, seemed to be lower in the Hydrus group than in the iStent group from POM1 to 12 but not at POM24. The iStent group also had more occurrences of significant BCVA worsening and need for additional IOP-lowering procedures, including glaucoma filtration surgery, deep sclerectomy, and selective laser trabeculoplasty, than the Hydrus group, although BCVA was overall significantly better in the iStent group from POM6 onwards.

Compared to outcomes reported in the existing literature, our results suggest lower efficacy of trabecular bypass MIGS in HM eyes compared with that in non-HM eyes. Existing landmark multicenter randomized controlled trials in non-HM eyes report higher rates of patients achieving complete success criteria at 2 years or more, although these studies included a washout period for glaucoma medication at baseline. Samuelson et al.’s study on the iStent inject and the HORIZON trial on the Hydrus microstent reported 63.2% and 49.5% of POAG subjects achieving an IOP of ≤ 18 mmHg without anti-glaucoma drops 2 years after iStent inject and 5 years after Hydrus microstent implantation, respectively, with 84% and 78% of subjects attaining drop-freedom 2 years after iStent inject and Hydrus microstent implantation, respectively [23, 24]. iStent and Hydrus implantation in both studies appear to demonstrate superior efficacy compared with that in our study, which had a smaller proportion of subjects attaining ≤ 18 mmHg without anti-glaucoma drops (iStent: 31.7%; Hydrus: 43.3%) and drop-freedom (iStent: 38.5%; Hydrus: 48.7%) at 24 months. However, direct comparison with non-HM cohorts from existing literature is limited by heterogeneity in study populations, surgical protocols, and outcome definitions. A randomized controlled trial with a concurrent control group would be required to definitively establish differences in MIGS efficacy between HM and non-HM eyes.

A few factors may contribute to the apparent lower IOP-lowering efficacy of both trabecular bypass MIGS devices in our study population. Firstly, our study had a relatively low baseline medicated IOP. The baseline medicated IOP in our study was lower than that in Samuelson et al. (15.49 ± 0.46 mmHg vs. 17.54 ± 2.99 mmHg) and in the HORIZON study (15.19 ± 0.46 mmHg vs. 17.9 ± 3.1 mmHg) [23, 24]. The low baseline medicated IOP, in addition to the absence of a medication washout period, is likely to have resulted in an overall lower IOP-lowering effect after MIGS [25, 26]. Secondly, our study had a greater proportion of patients with moderate than with mild POAG. The lower baseline VF MD in our study indicates more advanced disease than that in Samuelson et al. (– 6.12 ± 0.51 dB vs. −3.4 ± 3.3 dB) and in the HORIZON study (– 8.48 ± 0.50 dB vs. − 3.61 ± 2.49 dB) [23, 24]. A study cohort with more advanced disease may prompt lower IOP targets than one with milder disease. This may explain the lower baseline medicated IOP and lower proportions of patients achieving drop-freedom observed in our cohort. Of note, however, VF defects in HM glaucomatous eyes may be confounded by non-glaucomatous features of high myopia, including physiological blind spot enlargement from peripapillary atrophy and tilted discs, and myopia-associated temporal defects [27, 28]. Indeed several studies have demonstrated that myopic papillary and parapapillary deformations and deep-layer microvasculature dropout may influence the severity of glaucomatous VF defects and result in worse VF parameters in HM eyes with open-angle glaucoma (OAG) [29, 30]. Additionally, myopic macular degeneration, which was found in six eyes (4.69%) and nine eyes (7.26%) in the iStent and Hydrus groups, respectively, along with other retinal pathology, may also have confounded VF results.

Our results demonstrate low complication rates of trabecular bypass MIGS, even in HM eyes. At 2 years, Samuelson et al. reported that 3.9%, 1.8% and 5.4% of iStent inject implantations were complicated by hyphema, PAS, and secondary surgical intervention respectively [23]. The HORIZON trial reported that 1.4%, 5.9%, 10.6% and 0.3% of Hydrus microstent implantations were complicated by significant BCVA reduction, inflammation, PAS, and secondary surgical intervention, respectively, after 2 years [24]. PAS represent a significant mechanism of MIGS failure via chronic angle closure and aqueous outflow obstruction, and are frequently overlooked late complications that compromise long term surgical success after MIGS [31]. In contrast, our study showed that up to 24 months after MIGS implantation, only two eyes in the iStent group (one eye requiring filtration surgery [0.91%] at POM1, one eye requiring deep sclerectomy [0.88%] at POM12) and no eyes in the Hydrus group required secondary surgical intervention; no hyphema, inflammation, or PAS was reported in the iStent or Hydrus groups after 2 years. Our findings suggest that trabecular bypass MIGS may have similar safety profiles in POAG eyes with or without high myopia. This is despite anatomical differences that have been previously theorized to result in poor intraoperative visualization or malpositioning of trabecular MIGS devices.

At 2 years, 60.5% and 53.8% of eyes in the Hydrus and iStent groups, respectively, met the criteria for surgical success based on international guidelines. Both devices fell short of the 65% MCID threshold proposed by the AAO Preferred Practice Pattern Committee for MIGS combined with cataract surgery [19]. Notably, the proposed MCID thresholds were established by comparing MIGS, which offer superior safety profiles, against published success rates of trabeculectomies or tube shunt surgeries (82.7%–96.1% at 1 year), which have higher complication rates [32]. The same thresholds were also compared with standalone cataract surgeries (46%–61.9% at 2 years) [19]. In HM eyes, incisional glaucoma surgery such as trabeculectomy and tube shunt surgery may carry increased surgical risk and reduced efficacy, and decreased scleral rigidity also predisposes such eyes to hypotony-related complications [33]. Abe et al. reported higher trabeculectomy failure rates in HM than in non-HM eyes, although the difference did not reach statistical significance (37% vs. 22%, *P* = 0.067) [13]. Given the increased complication rates and lower success rates of traditional glaucoma surgery in HM eyes, trabecular bypass MIGS may remain a clinically relevant and safer alternative in these eyes with POAG, despite a lower proportion of patients reaching MCID thresholds.

Our results appear similar to those of studies comparing outcomes of non-trabecular bypass MIGS, such as ab-interno trabeculotomy, in high myopes and non-high myopes. Yoshida et al. reported that the probability of subjects with OAG achieving qualified success criteria of ≥ 20% IOP reduction from baseline, with or without anti-glaucoma eyedrops, was 65% in non-high myopes compared with 35% in high myopes at 24 months after ab-interno trabeculotomy (*P* = 0.038; log-rank test), with no significant difference in the incidence of postoperative complications (*P* = 1.0) [34]. Our findings similarly suggest reduced efficacy of MIGS in HM eyes.

Several possible theories related to the anatomical configuration of HM eyes may explain the limited effectiveness of trabecular bypass MIGS in these eyes. Studies have shown that high myopia is associated with larger SC areas, which are negatively correlated with IOP [16]. In contrast, POAG is associated with smaller SC areas, which are correlated with higher IOP [35], due to increased trabecular outflow resistance [36]. This may suggest that in HM eyes with POAG or OHT, the main point of resistance to aqueous outflow may occur distal to the SC. Thus, trabecular bypass MIGS may be insufficient to significantly lower IOP in HM eyes. Interestingly, a larger SC area has also been shown to result in greater IOP reduction with IOP-lowering agents [37]. In the context of ab-interno trabeculotomy, Yoshida et al. posited a few possible explanations behind its seemingly poorer efficacy in HM eyes. Firstly, irregular eye shapes may lead to obstructions in intrascleral collector channels and subsequent increased outflow resistance posterior to the SC; secondly, collagen fibre changes may cause functional outflow failure in intrascleral collector channels; and thirdly, altered cytokine and metabolite pathways may contribute to postoperative scarring in the trabecular meshwork and inhibit aqueous outflow [34]. These theories remain to be definitively proven.

Our study is limited by its retrospective nature. Postoperative data collection could only be meaningfully standardized at postoperative visits scheduled during POM1, 6, 12 and 24 timepoints. We were hence unable to conduct closer longitudinal monitoring to detect the exact date of failure for more accurate cumulative risk analysis. The retrospective design of our study also resulted in a fixed sample size without control over recruitment; hence, we did not conduct an a priori sample size calculation. Furthermore, our findings may be influenced by residual confounding from unmeasured variables and from time-varying confounding not captured in a static analysis, as well as by information bias arising from misclassification and measurement error. We also did not include interaction terms, so potential effect modification across subgroups was not assessed. We did not perform post hoc power calculations, as these are derived directly from the observed results and provide no information beyond the reported confidence intervals. Preoperative medicated IOP measurements were also analyzed without any washout period, which may have led to inaccuracies due to variable compliance and medication response. In the Hydrus group, patients were recruited from a single center, where most surgeries were performed by two surgeons, while patients in the iStent group were recruited predominantly from one of the three centers. This introduces potential center-specific bias related to patient selection, referral patterns, and institutional protocols, as well as surgeon-specific bias related to individual technique and treatment selection. Differences between groups may partly reflect center- or surgeon-level effects, and should be viewed as exploratory until confirmed in balanced multicenter studies. Eyes that had undergone standalone MIGS were not evaluated separately due to the low proportion involved in our study (3.9% iStent, none in the Hydrus group). These factors may lead to overestimation of success rates and underestimation of complication rates. Complication rates were also low, at ≤ 5 events per complication, which may suggest that our study was underpowered for reliable estimation and comparison of complication rates between the groups.

## Conclusion

In conclusion, our study demonstrates similar rates of both complete and qualified success between the Hydrus microstent and iStent inject in HM eyes up to 2 years postoperatively. In this HM-only cohort, IOP reduction and rates of drop-freedom after trabecular bypass MIGS appeared lower than those reported in non-HM eyes in the existing literature, although direct comparative studies are needed to evaluate this association. Both trabecular bypass MIGS devices demonstrated good safety throughout the follow-up duration. The findings of our study may better inform clinicians regarding the expected extent of IOP reduction and drop-freedom following trabecular bypass MIGS in HM eyes. More research into IOP-lowering options for the HM eye is required, considering its unique anatomy and disease profile.

## Supplementary Information

Additional file 1.

Additional file 2.

## Data Availability

The datasets used and/or analysed during the current study are available from the corresponding author on reasonable request.

## Abbreviations

AAO: American Academy of Ophthalmology
AGM: Anti-glaucoma medication
BCVA: Best-corrected visual acuity
HM: Highly myopic
IOP: Intraocular pressure
MCID: Minimal clinically important difference
MD: Mean deviation
MIGS: Minimally invasive glaucoma surgery
OAG: Open-angle glaucoma
OHT: Ocular hypertension
OR: Odds ratio
PAS: Peripheral anterior synechiae
POAG: Primary open-angle glaucoma
POM: Postoperative month
SC: Schlemm’s canal
VF: Visual field
