# Supplementary material for: Surgical outcomes of trabecular micro-bypass minimally invasive glaucoma surgery devices in eyes with high myopia: a 24-month retrospective multicenter cohort study
Source: Eye Vis (Lond). 2026 Sep 29;13:40. doi: 10.1186/s40662-026-00512-1 (PMC13621464; doi:10.1186/s40662-026-00512-1)
Supplement: Supplementary file 1 [file 40662_2026_512_MOESM1_ESM.docx]

**Supplementary Table 1.1** Complete success—multivariable model, POM1 (non-converged)

| **Parameter** | **B (95% CI)** | **Exp(B), OR (95% CI)** | ***P* value** |
| --- | --- | --- | --- |
| (Intercept) | 2.307 (−0.21 to 4.83) | 10.048 (0.81 to 125.02) | 0.073 |
| Eye (OS vs. OD) | −0.317 (−0.92 to 0.28) | 0.728 (0.40 to 1.33) | 0.3 |
| Eye (OD) | 0 (reference) | 1 (reference) | — |
| Gender (female vs. male) | −0.016 (−0.65 to 0.62) | 0.984 (0.52 to 1.86) | 0.961 |
| Gender (male) | 0 (reference) | 1 (reference) | — |
| Ethnicity (others vs. Chinese) | 30.121^a^ | 12059820631774.7 (NA) | NA |
| Ethnicity (Malay vs. Chinese) | −0.625 (−2.93 to 1.69) | 0.535 (0.05 to 5.39) | 0.596 |
| Ethnicity (Indian vs. Chinese) | 1.145 (0.22 to 2.07) | 3.143 (1.25 to 7.91) | 0.015 |
| Ethnicity (Caucasian vs. Chinese) | 3.708 (1.68 to 5.74) | 40.779 (5.38 to 309.40) | 0.0003 |
| Ethnicity (Chinese) | 0 (reference) | 1 (reference) | — |
| Standalone surgery (yes vs. no) | 29.307^a^ | 5341715866044.0 (NA) | NA |
| Standalone surgery (No.) | 0 (reference) | 1 (reference) | — |
| Age | −0.039 (−0.08 to 0.00) | 0.962 (0.93 to 1.00) | 0.042 |
| Reduction in IOP from baseline of ≥ 20% without drops, POM1 (yes vs. no) | −0.726 (−1.48 to 0.03) | 0.484 (0.23 to 1.03) | 0.059 |
| Reduction in IOP from baseline of ≥ 20% without drops, POM1 (No.) | 0 (reference) | 1 (reference) | — |

Multivariable logistic regression (dependent variable: device group; iStent = reference). Model covariates: operative eye, gender, ethnicity, standalone surgery, age and the success outcome at the stated timepoint. ^a^ Hessian matrix singularity caused by this parameter. The model did not converge (singular Hessian matrix); validity of the fit is uncertain and the affected estimates are not interpreted. Reference categories are shown for completeness.

CI = confidence interval; IOP = intraocular pressure; OR = odds ratio; POM = postoperative month

**Supplementary Table 1.2**. Complete success—multivariable model, POM6 (non-converged).

| **Parameter** | **B (95% CI)** | **Exp(B), OR (95% CI)** | ***P* value** |
| --- | --- | --- | --- |
| (Intercept) | 3.207 (0.33 to 6.09) | 24.715 (1.39 to 439.05) | 0.029 |
| Eye (OS vs. OD) | −0.105 (−0.75 to 0.54) | 0.901 (0.47 to 1.72) | 0.75 |
| Eye (OD) | 0 (reference) | 1 (reference) | — |
| Gender (female vs. male) | −0.201 (−0.89 to 0.48) | 0.818 (0.41 to 1.62) | 0.564 |
| Gender (male) | 0 (reference) | 1 (reference) | — |
| Ethnicity (others vs. Chinese) | 30.125^a^ | 12108460833730.0 (NA) | NA |
| Ethnicity (Malay vs. Chinese) | −0.692 (−2.64 to 1.25) | 0.501 (0.07 to 3.51) | 0.486 |
| Ethnicity (Indian vs. Chinese) | 0.856 (−0.13 to 1.85) | 2.353 (0.88 to 6.33) | 0.09 |
| Ethnicity (Caucasian vs. Chinese) | 4.028 (1.95 to 6.11) | 56.154 (7.00 to 450.78) | 0.0001 |
| Ethnicity (Chinese) | 0 (reference) | 1 (reference) | — |
| Standalone surgery (yes vs. No) | 29.242^a^ | 5007806563635.5 (NA) | NA |
| Standalone surgery (No.) | 0 (reference) | 1 (reference) | — |
| Age | −0.054 (−0.10 to −0.01) | 0.948 (0.91 to 0.99) | 0.014 |
| Reduction in IOP from baseline of ≥ 20% without drops, POM6 (yes vs. no) | −0.563 (−1.41 to 0.28) | 0.570 (0.24 to 1.33) | 0.193 |
| Reduction in IOP from baseline of ≥ 20% without drops, POM6 (No.) | 0 (reference) | 1 (reference) | — |

Multivariable logistic regression (dependent variable: device group; iStent = reference). Model covariates: operative eye, gender, ethnicity, standalone surgery, age and the success outcome at the stated timepoint. ^a^ Hessian matrix singularity caused by this parameter. The model did not converge (singular Hessian matrix); validity of the fit is uncertain and the affected estimates are not interpreted. Reference categories are shown for completeness.

CI = confidence interval; IOP = intraocular pressure; OR = odds ratio; POM = postoperative month

**Supplementary Table 1.3** Complete success—multivariable model, POM12 (non-converged).

| **Parameter** | **B (95% CI)** | **Exp(B), OR (95% CI)** | ***P* value** |
| --- | --- | --- | --- |
| (Intercept) | 2.607 (−0.25 to 5.47) | 13.562 (0.78 to 236.50) | 0.074 |
| Eye (OS vs. OD) | −0.226 (−0.89 to 0.44) | 0.798 (0.41 to 1.55) | 0.506 |
| Eye (OD) | 0 (reference) | 1 (reference) | — |
| Gender (female vs. male) | 0.371 (−0.36 to 1.10) | 1.449 (0.70 to 3.01) | 0.319 |
| Gender (male) | 0 (reference) | 1 (reference) | — |
| Ethnicity (others vs. Chinese) | 29.566^a^ | 6923793459041.1 (NA) | NA |
| Ethnicity (Malay vs. Chinese) | −29.361^a^ | 1.77e−13 (NA) | NA |
| Ethnicity (Indian vs. Chinese) | 0.803 (−0.25 to 1.86) | 2.233 (0.78 to 6.41) | 0.135 |
| Ethnicity (Caucasian vs. Chinese) | 3.863 (1.83 to 5.90) | 47.620 (6.24 to 363.31) | 0.0002 |
| Ethnicity (Chinese) | 0 (reference) | 1 (reference) | — |
| Standalone surgery (yes vs. no) | 29.028^a^ | 4044690889215.2 (NA) | NA |
| Standalone surgery (No.) | 0 (reference) | 1 (reference) | — |
| Age | −0.044 (−0.08 to 0.00) | 0.957 (0.92 to 1.00) | 0.041 |
| Reduction in IOP from baseline of ≥ 20% without drops, POM12 (yes vs. no) | 0.099 (−0.82 to 1.02) | 1.104 (0.44 to 2.76) | 0.832 |
| Reduction in IOP from baseline of ≥ 20% without drops, POM12 (No.) | 0 (reference) | 1 (reference) | — |

Multivariable logistic regression (dependent variable: device group; iStent = reference). Model covariates: operative eye, gender, ethnicity, standalone surgery, age and the success outcome at the stated timepoint. ^a^ Hessian matrix singularity caused by this parameter. The model did not converge (singular Hessian matrix); validity of the fit is uncertain and the affected estimates are not interpreted. Reference categories are shown for completeness.

CI = confidence interval; IOP = intraocular pressure; OR = odds ratio; POM = postoperative month

**Supplementary Table 1.4** Complete success—multivariable model, POM24 (non-converged).

| **Parameter** | **B (95% CI)** | **Exp(B), OR (95% CI)** | ***P* value** |
| --- | --- | --- | --- |
| (Intercept) | 5.893 (1.64 to 10.15) | 362.619 (5.13 to 25621.48) | 0.007 |
| Eye (OS vs. OD) | −0.434 (−1.36 to 0.50) | 0.648 (0.26 to 1.64) | 0.361 |
| Eye (OD) | 0 (reference) | 1 (reference) | — |
| Gender (female vs. male) | −1.078 (−2.15 to −0.01) | 0.340 (0.12 to 0.99) | 0.048 |
| Gender (male) | 0 (reference) | 1 (reference) | — |
| Ethnicity (others vs. Chinese) | 1.412 (−0.82 to 3.64) | 4.104 (0.44 to 38.20) | 0.215 |
| Ethnicity (Malay vs. Chinese) | −0.228 (−1.67 to 1.22) | 0.796 (0.19 to 3.37) | 0.757 |
| Ethnicity (Indian vs. Chinese) | 33.091^a^ | 235013620126980.0 (NA) | NA |
| Ethnicity (Caucasian vs. Chinese) | 0 (reference) | 1 (reference) | — |
| Standalone surgery (No., ref) | 0 (reference) | 1 (reference) | — |
| Age | −0.090 (−0.15 to −0.03) | 0.913 (0.86 to 0.97) | 0.004 |
| Reduction in IOP from baseline of ≥ 20% without drops, POM24 (yes vs. no) | −1.898 (−4.60 to 0.80) | 0.150 (0.01 to 2.23) | 0.168 |

Multivariable logistic regression (dependent variable: device group; iStent = reference). Model covariates: operative eye, gender, ethnicity, standalone surgery, age and the success outcome at the stated timepoint. ^a^ Hessian matrix singularity caused by this parameter. The model did not converge (singular Hessian matrix); validity of the fit is uncertain and the affected estimates are not interpreted. Reference categories are shown for completeness.

CI = confidence interval; IOP = intraocular pressure; OR = odds ratio; POM = postoperative month

**Supplementary Table 1.5** Qualified success—multivariable model, POM1 (non-converged).

| **Parameter** | **B (95% CI)** | **Exp(B), OR (95% CI)** | ***P* value** |
| --- | --- | --- | --- |
| (Intercept) | 2.288 (−0.24 to 4.81) | 9.852 (0.79 to 123.14) | 0.076 |
| Eye (OS vs. OD) | −0.330 (−0.93 to 0.27) | 0.719 (0.39 to 1.31) | 0.282 |
| Eye (OD) | 0 (reference) | 1 (reference) | — |
| Gender (female vs. male) | −0.031 (−0.67 to 0.61) | 0.969 (0.51 to 1.84) | 0.924 |
| Gender (male) | 0 (reference) | 1 (reference) | — |
| Ethnicity (others vs. Chinese) | 30.083^a^ | 11614778365748.9 (NA) | NA |
| Ethnicity (Malay vs. Chinese) | −0.648 (−2.98 to 1.68) | 0.523 (0.05 to 5.37) | 0.585 |
| Ethnicity (Indian vs. Chinese) | 1.190 (0.26 to 2.12) | 3.287 (1.30 to 8.35) | 0.012 |
| Ethnicity (Caucasian vs. Chinese) | 3.697 (1.67 to 5.72) | 40.318 (5.31 to 306.04) | 0.0003 |
| Ethnicity (Chinese) | 0 (reference) | 1 (reference) | — |
| Standalone surgery (yes vs. no) | 29.233^a^ | 4962420434906.6 (NA) | NA |
| Standalone surgery (No.) | 0 (reference) | 1 (reference) | — |
| Age | −0.038 (−0.08 to 0.00) | 0.962 (0.93 to 1.00) | 0.049 |
| Reduction in IOP from baseline of ≥ 20% with/without drops, POM1 (Yes vs. No) | −0.747 (−1.45 to −0.04) | 0.474 (0.24 to 0.96) | 0.037 |
| Reduction in IOP from baseline of ≥ 20% with/without drops, POM1 (No.) | 0 (reference) | 1 (reference) | — |

Multivariable logistic regression (dependent variable: device group; iStent = reference). Model covariates: operative eye, gender, ethnicity, standalone surgery, age and the success outcome at the stated timepoint. ^a^ Hessian matrix singularity caused by this parameter. The model did not converge (singular Hessian matrix); validity of the fit is uncertain and the affected estimates are not interpreted. Reference categories are shown for completeness.

CI = confidence interval; IOP = intraocular pressure; OR = odds ratio; POM = postoperative month

**Supplementary Table 1.6** Qualified success—multivariable model, POM6 (non-converged).

| **Parameter** | **B (95% CI)** | **Exp(B), OR (95% CI)** | ***P* value** |
| --- | --- | --- | --- |
| (Intercept) | 3.158 (0.25 to 6.07) | 23.533 (1.28 to 431.54) | 0.033 |
| Eye (OS vs. OD) | −0.109 (−0.75 to 0.53) | 0.897 (0.47 to 1.71) | 0.74 |
| Eye (OD) | 0 (reference) | 1 (reference) | — |
| Gender (female vs. male) | −0.205 (−0.88 to 0.48) | 0.814 (0.41 to 1.61) | 0.554 |
| Gender (male) | 0 (reference) | 1 (reference) | — |
| Ethnicity (others vs. Chinese) | 30.089^a^ | 11686388399204.5 (NA) | NA |
| Ethnicity (Malay vs. Chinese) | −0.713 (−2.66 to 1.24) | 0.490 (0.07 to 3.45) | 0.474 |
| Ethnicity (Indian vs. Chinese) | 0.879 (−0.10 to 1.86) | 2.408 (0.90 to 6.44) | 0.08 |
| Ethnicity (Caucasian vs. Chinese) | 4.065 (1.98 to 6.15) | 58.266 (7.27 to 466.86) | 0.0001 |
| Ethnicity (Chinese) | 0 (reference) | 1 (reference) | — |
| Standalone surgery (yes vs. no) | 29.319^a^ | 5410788348713.3 (NA) | NA |
| Standalone surgery (No.) | 0 (reference) | 1 (reference) | — |
| Age | −0.053 (−0.10 to −0.01) | 0.948 (0.91 to 0.99) | 0.016 |
| Reduction in IOP from baseline of ≥ 20% with/without drops, POM6 (yes vs. no) | −0.358 (−1.08 to 0.37) | 0.699 (0.34 to 1.45) | 0.334 |
| Reduction in IOP from baseline of ≥ 20% with/without drops, POM6 (No.) | 0 (reference) | 1 (reference) | — |

Multivariable logistic regression (dependent variable: device group; iStent = reference). Model covariates: operative eye, gender, ethnicity, standalone surgery, age and the success outcome at the stated timepoint. ^a^ Hessian matrix singularity caused by this parameter. The model did not converge (singular Hessian matrix); validity of the fit is uncertain and the affected estimates are not interpreted. Reference categories are shown for completeness.

CI = confidence interval; IOP = intraocular pressure; OR = odds ratio; POM = postoperative month

**Supplementary Table 1.7** Qualified success—multivariable model, POM12 (non-converged).

| **Parameter** | **B (95% CI)** | **Exp(B), OR (95% CI)** | ***P* value** |
| --- | --- | --- | --- |
| (Intercept) | 2.611 (−0.33 to 5.55) | 13.610 (0.72 to 257.50) | 0.082 |
| Eye (OS vs. OD) | −0.206 (−0.88 to 0.46) | 0.813 (0.42 to 1.59) | 0.545 |
| Eye (OD) | 0 (reference) | 1 (reference) | — |
| Gender (female vs. male) | 0.438 (−0.30 to 1.18) | 1.550 (0.74 to 3.24) | 0.244 |
| Gender (male) | 0 (reference) | 1 (reference) | — |
| Ethnicity (others vs. Chinese) | 29.314^a^ | 5382060573424.9 (NA) | NA |
| Ethnicity (Malay vs. Chinese) | −29.410^a^ | 1.69e−13 (NA) | NA |
| Ethnicity (Indian vs. Chinese) | 0.883 (−0.17 to 1.94) | 2.418 (0.84 to 6.95) | 0.101 |
| Ethnicity (Caucasian vs. Chinese) | 3.877 (1.85 to 5.90) | 48.261 (6.36 to 366.47) | 0.0002 |
| Ethnicity (Chinese) | 0 (reference) | 1 (reference) | — |
| Standalone surgery (yes vs. no) | 28.930^b^ | 3667222432089.0 (NA) | NA |
| Standalone surgery (No.) | 0 (reference) | 1 (reference) | — |
| Age | −0.046 (−0.09 to 0.00) | 0.955 (0.92 to 1.00) | 0.035 |
| Reduction in IOP from baseline of ≥ 20% with/without drops, POM12 (yes vs. no) | 0.644 (−0.15 to 1.43) | 1.904 (0.86 to 4.20) | 0.11 |
| Reduction in IOP from baseline of ≥ 20% with/without drops, POM12 (No.) | 0 (reference) | 1 (reference) | — |

Multivariable logistic regression (dependent variable: device group; iStent = reference). Model covariates: operative eye, gender, ethnicity, standalone surgery, age and the success outcome at the stated timepoint. ^a^ Hessian matrix singularity caused by this parameter. The model did not converge (singular Hessian matrix); validity of the fit is uncertain and the affected estimates are not interpreted. Reference categories are shown for completeness.

CI = confidence interval; IOP = intraocular pressure; OR = odds ratio; POM = postoperative month

**Supplementary Table 1.8** Qualified success—multivariable model, POM24 (non-converged).

| **Parameter** | **B (95% CI)** | **Exp(B), OR (95% CI)** | ***P* value** |
| --- | --- | --- | --- |
| (Intercept) | 5.683 (1.32 to 10.04) | 293.861 (3.76 to 22985.43) | 0.011 |
| Eye (OS vs. OD) | −0.411 (−1.31 to 0.49) | 0.663 (0.27 to 1.63) | 0.37 |
| Eye (OD) | 0 (reference) | 1 (reference) | — |
| Gender (female vs. male) | −1.027 (−2.08 to 0.03) | 0.358 (0.12 to 1.03) | 0.057 |
| Gender (male) | 0 (reference) | 1 (reference) | — |
| Ethnicity (others vs. Chinese) | 0.596 (−1.38 to 2.58) | 1.815 (0.25 to 13.15) | 0.555 |
| Ethnicity (Malay vs. Chinese) | −0.157 (−1.57 to 1.26) | 0.855 (0.21 to 3.52) | 0.828 |
| Ethnicity (Indian vs. Chinese) | 32.898^a^ | 193746400123860.0 (NA) | NA |
| Ethnicity (Caucasian vs. Chinese) | 0 (reference) | 1 (reference) | — |
| Standalone surgery (No., ref) | 0 (reference) | 1 (reference) | — |
| Age | −0.089 (−0.15 to −0.02) | 0.915 (0.86 to 0.98) | 0.006 |
| Reduction in IOP from baseline of ≥ 20% with/without drops, POM24 (yes vs. no) | −0.154 (−1.12 to 0.81) | 0.857 (0.33 to 2.26) | 0.755 |
| Reduction in IOP from baseline of ≥ 20% with/without drops, POM24 (No ) | 0 (reference) | 1 (reference) | — |

Multivariable logistic regression (dependent variable: device group; iStent = reference). Model covariates: operative eye, gender, ethnicity, standalone surgery, age and the success outcome at the stated timepoint. ^a^ Hessian matrix singularity caused by this parameter. The model did not converge (singular Hessian matrix); validity of the fit is uncertain and the affected estimates are not interpreted. Reference categories are shown for completeness.

CI = confidence interval; IOP = intraocular pressure; OR = odds ratio; POM = postoperative month

**Supplementary Table 2.** Case distribution by center

| **Center** | **iStent**  **(n = 128)** | **Hydrus**  **(n = 124)** |
| --- | --- | --- |
| Singapore National Eye Center, Singapore (n, %) | 51 (39.8%) | 124 (100%) |
| Sydney Eye Surgeons, Sydney, Australia (n, %) | 61 (47.7%) | - |
| Mahatma Eye Hospital, Trichy, Tamil Nadu, India (n, %) | 16 (12.5%) | - |

**Supplementary Table 3**: POM1–6 postoperation complications (iStent vs. Hydrus)

| **Outcome** | **POM1** | | **POM6** | |
| --- | --- | --- | --- | --- |
|  | **iStent**  **(n = 110)** | **Hydrus**  **(n = 124)** | **iStent**  **(n = 106)** | **Hydrus**  **(n = 110)** |
| **Post-op complications no. (%)** | | | | |
| Hyphema | 5 (4.54%) | 1 (0.81%) | 3 (2.83%) | 0 |
| IOP spike (> 6mm Hg from baseline) | 5 (4.54%) | 6 (4.84%) | 2 (1.89%) | 2 (1.82%) |
| IOP spike (> 30 mmHg) | 0 | 0 | 0 | 0 |
| IOP spike > 10 mmHg | 1 (0.91%) | 5 (4.03%) | 1 (0.94%) | 2 (1.82%) |
| PAS | 1 (0.91%) | 1 (0.81%) | 1 (0.94%) | 1 (0.91%) |
| Others | 1 Corneal blood staining (0.91%), 1 Pigmentary glaucoma (0.91%), 1 inflammation/ uveitis (0.91%) | 1 PCR (0.81%), 1 Toric IOL rotation (0.81%) | 2 inflammation/ uveitis (1.89%) | 0 |

POM = postoperative month; Post-op = postoperation; IOP = intraocular pressure; PAS = posterior anterior synechiae

**Supplementary Table 4**. POM12–24 postoperation complication rates (iStent vs. Hydrus)

| **Outcome** | **POM12** | | **POM24** | |
| --- | --- | --- | --- | --- |
|  | **iStent**  **(n = 114)** | **Hydrus**  **(n = 91)** | **iStent**  **(n = 65)** | **Hydrus**  **(n = 81)** |
| **Post-op complications no. (%)** | | | | |
| Hyphema | 0 | 0 | 0 | 0 |
| IOP spike (> 6 mmHg from baseline) | 0 | 1 (1.10%) | 1 (1.54%) | 2 (2.47%) |
| IOP spike (> 30 mmHg) | 0 | 0 | 0 | 0 |
| PAS | 1 (0.88%) | 1 (1.10%) | 0 | 0 |
| Others | 1 inflammation/ uveitis (0.88%) | 0 | 0 | 0 |

POM = postoperative month; Post-op = postoperation; IOP = intraocular pressure; PAS = posterior anterior synechiae

**Supplementary Table 5**. Postoperative BCVA (iStent vs. Hydrus)

| **BCVA**  **(LogMAR)** | **iStent**  **(mean ± SE)** | **Hydrus**  **(mean ± SE)** | **Mean difference**  **(mean ± SE)** | ***P* value** |
| --- | --- | --- | --- | --- |
| POM1 | 0.141 ± 0.021 | 0.138 ± 0.021 | 0.004 ± 0.027 | 0.893 |
| POM6 | 0.133 ± 0.020 | 0.188 ± 0.021 | −0.055 ± 0.024 | 0.022 |
| POM12 | 0.108 ± 0.019 | 0.200 ± 0.022 | −0.092 ± 0.025 | 0.000 |
| POM24 | 0.133 ± 0.026 | 0.241 ± 0.024 | −0.108 ± 0.035 | 0.002 |

POM = Postoperative month; BCVA = best-corrected visual acuity; SE = standard error; 95% CI = 95% = confidence interval
