# Supplementary material for: Surgical outcomes of trabecular micro-bypass minimally invasive glaucoma surgery devices in eyes with high myopia: a 24-month retrospective multicenter cohort study
Source: Eye Vis (Lond). 2026 Sep 29;13:40. doi: 10.1186/s40662-026-00512-1 (PMC13621464; doi:10.1186/s40662-026-00512-1)
Supplement: Supplementary file 2 [file 40662_2026_512_MOESM2_ESM.docx]

**Supplementary Figure 1.** Scatterplots of combined success criteria of ≤ 15 mmHg (dashed red line), ≤ 18 mmHg (dotted blue line), ≥ 20% IOP reduction (oblique black solid line) with a safety IOP threshold of ≤ 21mmHg (green solid line), with (blue dots) or without (red dots) use of anti-glaucoma eyedrops at POM1, 6, 12 and 24 (iStent versus Hydrus). IOP, intraocular pressure; POM, postoperative month.

|  |  |
| --- | --- |
|  |  |
|  |  |
|  |  |

**Supplementary Figure 2.** Scatterplot of baseline versus POM12 BCVA in logMAR units, with safety threshold of BCVA worsening by an additional 0.2 logMAR from baseline (black dashed line), iStent versus Hydrus. LogMAR conversions: Counting fingers = 1.8, Hand movements = 2.3. POM, postoperative month; BCVA, best-corrected visual acuity.
